# Complete mitochondrial genome of a Siberian Crane (*Grus leucogeranus*)

**DOI:** 10.1080/23802359.2018.1467224

**Published:** 2018-05-10

**Authors:** Tong Wang, Hongcheng Wang, Zhongbao Zhao, Zongtao Wang, Liqiang Mu, Hongxian Yu

**Affiliations:** a Horticultural Sub-Academy, Heilongjiang Academy of Agricultural Sciences, Harbin, China;; b School of Forestry, Northeast Forestry University, Harbin, China;; c College of Wildlife Resources, Northeast Forestry University, Harbin, China;; d Department of Ecology, Hebei University of Environmental Engineering, Qinhuangdao, China;; e Heilongjiang Academy of Agricultural Sciences, Harbin, China

**Keywords:** Complete mitogenome, Siberian Crane, high-throughput genetic sequencing

## Abstract

The complete mitochondrial genome (mtDNA) of hemocyte from Siberian Crane (*Grus leucogeranus*) was sequenced using high-throughput genetic sequencing, and mitochondrial genome was assembled using MITObim tools according complete mitochondrial genome of *Grus leucogeranus* (NCBI Reference Sequence: NC_020574.1). The assembled mitogenome, consisting of 16,747 bp, has unique 14 protein-coding genes (PCGs), 22 transfer RNAs, and two ribosomal RNAs genes. The complete mitogenome provides essential and important DNA molecular data for further phylogenetic and evolutionary analysis for Siberian Crane phylogeny.

Siberian Crane (*Grus leucogeranus*) is listed as a threatened species according to the IUCN ‘red list’ (Birdlife International). The global population of Siberian Cranes is estimated to be approximately 4000 individuals, which are separated into three recognized populations (i.e. eastern, central, and western) based on migration routes. The eastern population is the largest, accounting for approximately 99% of the total number (Li et al. 2012). In autumn, Siberian Cranes migrate from Northeast Siberia in Russia to Poyang Lake in the Yangtze River basin of China, and return to Siberia the following spring (Kanai et al. 2002; Jiang et al. 2015). Sequence information on the family Cranes of which the Siberian Crane is a member, is scant in the current databases and as such is inadequate for the purpose of performing extensive genomics based high throughput analyses to unravel information on phylogenetic relationships. Therefore, we determined the complete mitogenome sequence of the Siberian Crane in order to serve as a resource for research.

Peripheral blood of Siberian Crane (♀) were collected from Bei Fang Seng Lin Zoo in A’cheng district, Harbin, Heilongjiang Province (126.4200°N, 45.1200°E), China. The specimens were kept in the Feline Research Center of Chinese State Forestry Administration at −80 °C (No.Whc-G.Leu20171014a), total genomic DNA was extracted using a DNA extract kit following the previously reports (Gunal et al. 2018). And we used high-throughput genetic sequencing to perform low coverage whole genome sequencing. Initially, the raw high-throughput genetic sequencing reads generated from Illumina HiSeq PE150 (Illumina, San Diego, CA). 40,918,448 clean reads were de novo assembly by using commercial software (Geneious V9, Auckland, New Zealand) to produce a single, circular form of complete mitogenome. The total length of its mitogenome was 16,747 bp, with a genome size similar to other research (Krajewsk et al. 2010), but more details on that study. Compared with the current mitogenome sequences of Siberian Crane, more than 1400 bases substitution in the Siberian Crane mitogenome of China we sequenced. The accurate annotated mitochondrial genome sequence was submitted to GeneBank with accession number MH041490. The complete mitogenome of Siberian Crane was 16,747 bp in size and its overall base composition is 31.4% for A, 31.0% for C, 13.5% for G, and 24.1% for T, and have GC content of 43.97%.

The protein coding, rRNA and tRNA genes of Siberian Crane mitogenome were predicted by using DOGMA (Wyman et al. 2004), ARWEN (Laslett and Canback 2008), MITOS (Bernt et al. 2013) tools and manually inspected. The complete mitogenome of Siberian Crane includes unique 14 protein-coding genes (PCGs), 22 transfer RNA genes, and two ribosomal RNA genes, all details were listed in GeneBank (MH041490).

To further validate the new sequences, we used all of mitochondrial genome sequences published in Genebank of other crane species to construct the phylogenetic tree. These species were as follows: *Grus japonensis*, *Grus americana*, *Grus grus*, *Grus monacha*, *Grus nigricollis*, *Grus carunculatus*, *Anthropoides paradiseus*, *Grus vipio*, *Grus rubicunda*, *Grus antigone*, *Grus Canadensis*, and *Grus leucogeranus*. We used MEGA6 to produce the phylogenetic tree based on the maximum-likelihood method (Figure 1). The phylogenetic analysis results support that the mitochondrial DNAs of Siberian Crane are more closely related to other species of crane genus.

**Figure 1.**
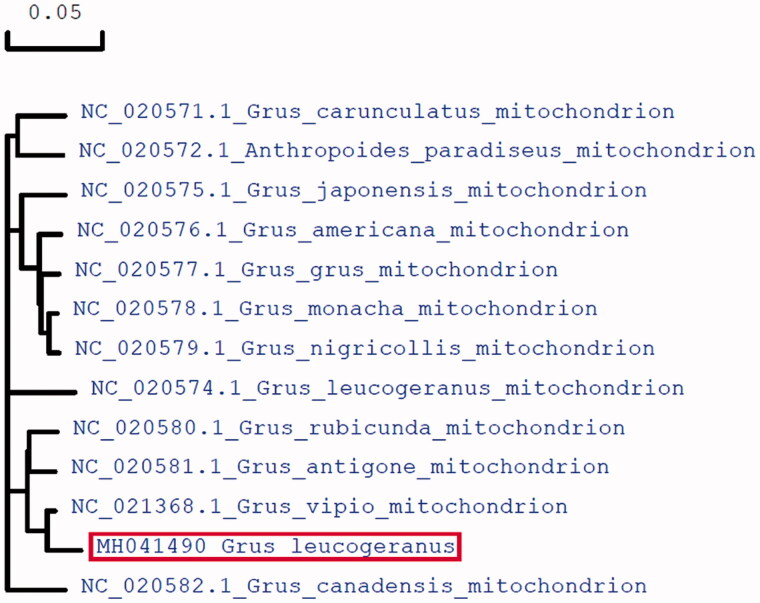
Molecular phylogeny of Siberian Crane and related species in Crane based on complete mitogenome. The complete mitogenomes are downloaded from GeneBank and the phylogenetic tree is constructed by maximum-likelihood method with 500 bootstrap replicates. The gene’s accession number for tree construction is listed in front of the species name.

## References

[CIT0007] Bernt M, Donath A, Juhling F, Externbrink F, Florentz G, Putz J, Middendorf M, Stadler PF. 2013 MITOS: improved de novo metazoanmitochondrial genome annotation. Mol Phylogenet Evol. 69:313–319. 22982435 10.1016/j.ympev.2012.08.023

[CIT0001] Birdlife International IUCN Red List of Threatened Species; [accessed 2013 Mar 10]. http://www.Iucnredlist.org.

[CIT0002] Krajewsk C, Sipiorsk JT, Anderson FE. 2010 Complete mitochondrial genome sequences and the phylogeny of Cranes (Gruiformes: Gruidae). Auk. 127:440–452.

[CIT0003] Gunal G, Kip C, Eda Ogut S, Ilhan H, Kibar G, Tuncel A. 2018 Comparative DNA isolation behaviours of silica and polymer based sorbents in batch fashion: monodisperse silica microspheres with bimodal pore size distribution as a new sorbent for DNA isolation. Artif Cells Nanomed Biotechnol. 46:178–184. 10.1080/21691401.2017.1304404 28328301

[CIT0004] Jiang H, He C, Sheng L, Tang Z, Wen Y, Yan T, Zou C. 2015 Hydrological modelling for Siberian Crane *Grus leucogeranus* stopover sites in Northeast China. PLoS One. 10:e0122687. 25874552 10.1371/journal.pone.0122687 PMC4398425

[CIT0005] Kanai Y, Ueta M, Germogenov N, Nagendran M, Mita N, Higuchi H. 2002 Migration routes and important resting areas of Siberian cranes (*Grus leucogeranus*) between northeastern Siberia and China as revealed by satellite tracking. Biol Conserv. 106:339–346.

[CIT0006] Li FS, Wu JD, Harris J, Burnham J. 2012 Number and distribution of cranes wintering at Poyang Lake, China during 2011–2012. Chin. Birds. 3:180–190.

[CIT0008] Laslett D, Canback B. 2008 ARWEN: a program to detect tRNA genes inmetazoan mitochondrial nucleotide sequences. Bioinformatics. 24:172–175. 18033792 10.1093/bioinformatics/btm573

[CIT0009] Wyman SK, Jansen RK, Boore JL. 2004 Automatic annotation of organellargenomes with DOGMA. Bioinformatics. 20:3252–3255. 15180927 10.1093/bioinformatics/bth352

